# Shuangshi Tonglin Capsule treats benign prostatic hyperplasia through the ROS/NLRP3 signaling pathway

**DOI:** 10.1007/s11255-023-03874-w

**Published:** 2023-12-01

**Authors:** Ziqiang Wang, Qian Mao, Yong Yuan, Chuan Wang, Hao Wei

**Affiliations:** School of Pharmacy, Shaanxi University of Chinese Medicine, Xianyang, 712046 Shaanxi China

**Keywords:** Shuangshi Tonglin Capsule, Benign prostatic hyperplasia, Oxidative stress, ROS, Oxidative stress, NLRP3

## Abstract

**Objective:**

To explore the effects of the SSTL on BPH and clarify the therapeutic mechanisms.

**Methods:**

Animal model of BPH was established by castration and subcutaneous injection of TP into SD rats; rats were orally administered SSTL for 28 days while modeling. Detection of PI, LI and RI in rats, to observe histopathological changes and collagen deposition in the prostate tissue. Detects levels of sex hormones and inflammatory factors in serum and tissues of rats, the test kit detects levels of lipid peroxides and antioxidants in serum and tissues. Fluorescent staining analysis of tissue ROS; the expression of NLRP3 inflammatory vesicles was observed by immunohistochemistry; Western blotting detected the expression of NOX4, NOX2, NLRP3 inflammatory vesicles, ASC, Cleaved Caspase-1, Caspase-1, IL-1β.

**Results:**

After SSTL capsule treatment, the PI and RI of the rats decrease. HE and Masson staining showed that SSTL ameliorated the pathological damage and reduced collagen deposition in the prostate tissue of BPH rats; ELISA results showed that SSTL was able to reduce T, DHT, TNF-α, IL-1β levels in BPH rats. The test kit showed that SSTL made the levels of MDA, CAT and GSH-Px in the serum and prostate tissue of rats and increased the activity of SOD. The results of ROS fluorescence showed that the ROS level was reduced in SSTL group; Western blotting showed that SSTL could cause down-regulation of NOX4, NOX2, NLRP3, ASC, Cleaved Caspase-1, IL-1β protein expression.

**Conclusion:**

SSTL can reduce the PI and RI in BPH rats, it can also inhibit the level of sex hormones and inflammatory factors in BPH rats, which thereby reducing the histopathological damage of prostate gland in BPH rats, and can treat BPH in rats through ROS/NLRP3 pathway.

**Supplementary Information:**

The online version contains supplementary material available at 10.1007/s11255-023-03874-w.

## Introduction

Benign prostatic hyperplasia (BPH) is a condition that is more common in middle-aged and older [1]. Along with the aging of the world population, the overall prevalence of BPH is on the rise, with the prevalence of BPH being about 40% for men over the age of 60 and 80% for men over the age of 90 [2]. BPH generally leads to lower urinary tract symptoms (LUTS) and bladder outlet obstruction (BOO). Its clinical manifestations specifically include frequent urination, increased frequency of nocturia, weakness of urination [3]. According to the histological diagnosis, BPH is characterized by the abnormal proliferation of prostate epithelial cells and stromal cells [4]. The pathogenesis of BPH has not been fully elucidated in modern medicine, However, studies have pointed to a possible link with the imbalance of proliferation and apoptosis of epithelial and mesenchymal cells. The pathogenesis may be related to sex hormones, immune inflammation, growth factors, other factors [5].

Oral chemotherapy is widely used in the treatment of BPH, it mainly includes α-blockers, 5α-reductase inhibitors and phytotherapeutic agents [6]. However, different types of chemical drugs often bring side effects. α1-blockers can be associated with gastrointestinal reactions and upright hypotension [7], 5α-reductase inhibitors can be associated with sexual dysfunction, which can lead to decreased libido, ejaculation disorders, and erectile dysfunction [8]. The use of herbal medicine to alleviate BPH has received increasing attention in recent years in clinical practice.

Shuangshi Tonglin Capsule (SSTL) is a standardized Chinese herbal preparation developed from the prescription of “Decoction Chengshi Bixie Fengqing”. The prescription originated from the Qing Dynasty and contains 10 traditional Chinese herbs (Table 1). The preparation has a long history of application and is highly effective in treating chronic prostatitis and prostate enlargement [9].

**Table 1:** Composition of SSTL

Number	Chinese name	English name	Plant part
1	Fengbixie	*Dioscorea polystachya* Turcz	Root
2	Guanhuangbai	*Phellodendron amurense* Rupr	Bark
3	Baijiangcao	*Patrinia scabiosifolia Fisch*. ex Trevir	Whole herb
4	Qingdai	*Isatis tinctoria* L	Aerial part
5	Huashi	*Talcum*	Mineral
6	Cheqianzi	*Plantago asiatica* L	Seed
7	Shichangpu	*Acorus calamus* L	Whole herb
8	Fuling	*Wolfiporia cocos* (F.A.Wolf) Ryvarden & Gilb	Root
9	Cangzhu	*Atractylodes lancea* (Thunb.) DC	Root
10	Danshen	*Salvia miltiorrhiza* BungeBPH mo	Root

The pathogenesis of BPH is unclear but oxidative stress and inflammation have been shown to be strongly associated with the pathogenesis of BPH [10]. Oxidative stress is an imbalance between the production of oxidants and the antioxidant capacity, which in turn causes the production of reactive oxygen species (ROS) [12]. NADPH oxidase 4 (NOX4) has been described as a novel source of ROS induction in macrophages. A Studies have reported that NOX4-derived ROS can promote BPH, at the same time, ROS can cause cellular damage through multiple pathways, including the release of pro-inflammatory cytokines, the depletion of antioxidants and the activation of pro-inflammatory signaling pathways [13, 14].

Numerous studies have also shown that inflammation and metabolic syndrome (Mets) are inextricably linked to the development of BPH [15]. In the study, the inflammatory vesicles is responsible for responding to both endogenous and exogenous stress signals, which in turn initiate the inflammatory cascade response. It has been shown that the expression of mRNA encoding inflammatory vesicle components is higher in prostate biopsies from BPH patients compared to prostate cancer patients [16, 17]. NOD-like receptor thermal protein domain associated protein 3 (NLRP3) inflammatory vesicles are composed of NLRP3, Apoptosis-associated speck-like protein containing a CARD (ASC) and Cysteinyl aspartate specific proteinase-1 (Caspase-1). This inflammatory vesicle causes upregulation of Pro-Caspase-1 and NLRP3-related precursor expression mainly through the Nuclear factor kappa-B (NF-κB) signaling pathway, followed by assembly and activation in the presence of activators [18].

Oxidative stress injury and activation of NLRP3 inflammatory vesicles play an important role in the development of fibrotic disease. It has been shown that inflammatory vesicles and their downstream inflammatory factors Interleukin 18 (IL-18) and Interleukin 1β (IL-1β) are expressed in prostate tissue. Increased ROS is also a classical mechanism that induces NLRP3 inflammatory vesicle activation and plays an important role in fibrotic diseases. Therefore, reducing the inflammatory response and oxidative stress is one of the effective ways to treat BPH [19].

## Materials and methods

### Drug preparation

Shuangshi Tonglin Capsule (SSTL) contains a variety of herbal ingredients as shown in (Table 1), SSTL is manufactured by Shaanxi Momentum Pharmaceutical Co., Ltd. (Shaanxi, China). The China National Drug Administration (CNDA) number is Z20080028. Finasteride Tablets are manufactured by MSD Pharma (Singapore) Pte. Ltd. Preparation of SSTL suspension: Weigh the SSTL powder with 0.5% CMC-Na and keep grinding, It was prepared as a suspension with concentrations of SSTL low dose (0.0625 g/mL), SSTL medium dose (0.125 g/mL) and SSTL high dose (0.25 g/mL). Configuration of finasteride tablet suspensions: The finasteride tablet coating was scraped off with a knife, crushed in a mortar and weighed accurately, and 0.5% CMC-Na was added to form a suspension with a concentration of 0.052 mg/mL.

Preparation of mold-making agents: Testosterone propionate (TP) (MedChamExpress Cat.#HY-B1269/CS-4905) was dissolved in corn oil (Solarbio China No. C7030) and configured as 5 mg/ml TP solution.

### Animals and treatment

66 male SD rats were randomly divided into sham-operated group, model group, SSTL low dose, SSTL medium dose, SSTL high dose group, and positive drug group. SD rats were obtained from Chengdu Dashuo Experimental Animal Co., Ltd (Sichuan, China; Certificate of Conformity: SCXK (Chuan) 2020–030). Experiments were performed in accordance with the guidelines of the Animal Ethics Committee of Shaanxi University of Traditional Chinese Medicine (license number SUCMDL20220401002). Animal grouping and treatment:

- Sham group: Subcutaneous injection of equal amount of corn oil solution.
- Model group: Subcutaneous injection of TP (No.hy-b1269 Shanghai Haoyuan Biomedical Technology Co.) corn oil solution (5 mg/kg, 1 ml/1 kg).
- SSTL Low dose group (SSTL-L): SSTL suspension (0.625 g/kg, 1 ml/kg).
- SSTL Medium dose group (SSTL-M): SSTL suspension (1.25 g/kg, 1 ml/kg).
- SSTL High dose group (SSTL-H): SSTL suspension (2.5 g/kg, 1 ml/kg).
- Positive drug group (Fina): Finasteride (Fina) tablet suspensions (0.52 mg/kg, 1 ml/kg).

Subcutaneous injection and gavage were administered once a day for 28 days, the rats were weighed every 3 days to regulate the dosage of the drug. SD rats were euthanized after 28 days, and test samples were taken for subsequent testing.

**Figure:**
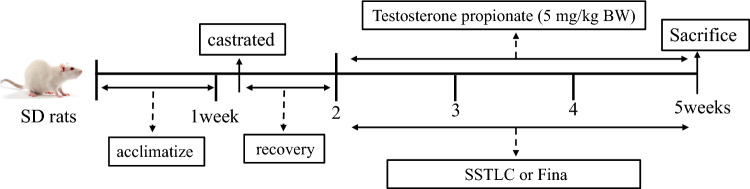
Animal experiment flowchart

### Detection of Organ index, body weight of rats and prostatic morphology

The rats in each group were weighed before anesthesia and the organ indices were calculated for each group according to the following formula:

$${\text{Prostate index }}\left( {PI} \right)\, = \,{\text{prostate weight }}\left( {mg} \right)/{\text{body weight }}(g)$$

$${\text{Liver index }}\left( {LI} \right)\, = \,{\text{liver weight }}\left( {mg} \right)/{\text{body weight }}(g)$$

$${\text{Renal index }}\left( {RI} \right)\, = \,{\text{renal weight}}\left( {mg} \right)/{\text{body weight }}(g)$$

### Masson’s trichrome staining

The 5 μm sections of` prostatic tissues were deparaffinized in xylene (three washes for 3 min each) and then hydrated in graded ethanol to distilled water. Slides were stained with a Masson’s trichrome-staining kit from ZSGB-BIO (Beijing, China), following the manufacturer’s protocol.

### Hematoxylin and eosin (H&E) staining

After collection, rat prostates were dewaxed in water, embedded, and cut into 5 µm sections and stained with H&E. Histopathological changes in the prostate were evaluated by microscopy.

### Analysis of T, DHT, TNF-α and IL-1β levels in serum and tissue

The ELISA method was used to determine the level of the Testosterone (T), Dihydrotestosterone (DHT), Tumor Necrosis Factor alpha (TNF-α) and IL-1β in serum and prostate tissue according to the manufacturer’s instructions (Shanghai Enzyme Biotechnology Co., Ltd. Shanghai, China, #ml002998, #ml002868).

### Detection of antioxidant enzyme activity and lipid peroxidation products

Prepare prostate tissue supernatant, determine the concentration, and place on ice with serum for use. The effects of antioxidant enzyme activities and lipid peroxidation products in prostate tissue and serum were examined according to superoxide dismutase (SOD), Catalase (CAT), glutathione peroxidase (GSH-PX), and Malondialdehyde (MDA) instructions.

### ROS staining of prostate tissue was observed by frozen section

The prostate tissue was embedded in OCT, snap frozen in liquid nitrogen to 80 °C, and the frozen tissue was then cut into sections of 5 μm thickness, added with autofluorescence quencher, dripped with ROS stain, washed sections, dripped with DAPI stain, washed sections and finally sealed. Photographs were taken using a fluorescence microscope (× 400), and the fluorescence intensity was analyzed using Image J image analysis software.

### Immunohistochemistry analysis

Immunohistochemistry (IHC) analysis was used to detect the expression of NLRP3 prostate tissue. The prostate tissue sections were dewaxed in water and the sections were washed with PBS (pH = 7.4). After washing, the tissue sections were treated with 3% H_2_O_2_ solution for 25 min at room temperature. After blocking, the sections were incubated overnight at 4 °C. Then, AEC color solution and hematoxylin were used for redyeing. Five randomly distributed fields within the prostate lobe on each slide were analyzed and cells with red granules were considered positive. Each slide was selected for counting NLRP3 positive cells.

### Western blot analysis

Total proteins were extracted from prostate tissues with RIPA buffer. Equal amounts of protein samples were loaded onto 10% SDS-PAGE gels and then transferred onto PVDF membranes. The PVDF membranes were blocked with 5% non-fat milk for 2 h at room temperature and then incubated with anti-IKKβ (1:1000, No. #8943), anti-P38(1:1000, No. #8690), anti-phospho-P38(1:1000, No. #4511), anti-ERK1/2(1:1000, No. #4695), anti-phospho-ERK1/2 (1:1000, No. #4370), anti-JNK/SAPK (1:1000, No. #9252), anti-phospho-JNK/SAPK (1:1000, No. #4668), anti-AMPK(1:1000, No. bs-10344R),anti-phospho-AMPK(1:1000, No. 2537S), anti-SIRT-1 (1:1000, No. ab110304), and anti-GAPDH (1:1000, No. GB11002) overnight at 4℃. The next day, the PVDF membranes were incubated with a secondary antibody for 2 h at room temperature. The protein signals were visualized with an enhanced chemiluminescence system and detected by ImageJ software. All protein signals were standardized by using GAPDH.

### Statistical analysis

All the data were analyzed with SPSS 26.0, and the results are expressed as the mean ± standard error of the mean (mean ± SD). Multiple comparisons were performed using one-way analysis of variance (ANOVA), and *P* < 0.05 or *P* < 0.01 were considered statistically significant. Statistical analyses were performed using GraphPad Prism 8.0 software (GraphPad Software, Inc., CA, USA).

## Results

### Effect of SSTL on body weight, prostate morphology and organ index in BPH rats

**Fig. 1:**
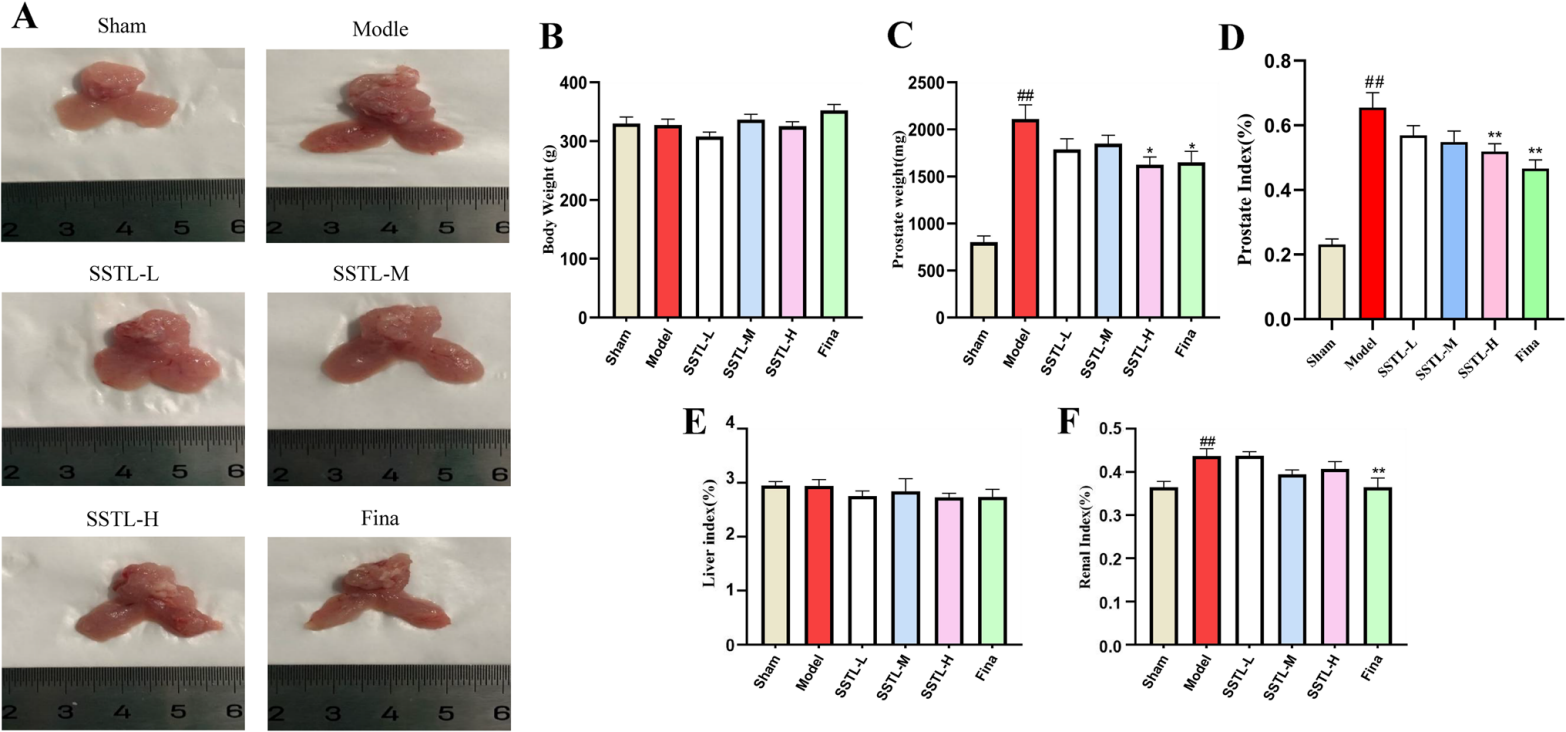
Effect of SSTLC on body weight, prostate morphology and organ index in BPH rats. **A** Prostate morphology; **B** Rat body weight; **C** Rat prostate wet weight; **D** Prostate index; **E** Liver index; **F** Renal index. Values are presented as means ± SEMs. #*P* < 0.05, ##*P* < 0.01 compared with the Sham group; *P < 0.05, **P < 0.01 compared the Model group (*n* = 6)

Observation of prostate morphology after 28 days of modeling and drug administration. The prostate of the Model group rats was significantly enlarged and hard, while the prostates of all groups decreased in size and normalized in shape. (Fig. 1A). There was no significant difference in body weight between groups of rats (Fig. 1B). The prostate index is considered one of the important biomarkers of BPH. Prostate wet weight and PI of rats in the Model group were significantly increased, (*P* < 0.01) the SSTL-H group and the Fina group significantly reduced prostate wet weight and PI. (*P* < 0.05 or *P* < 0.01) (Fig. 1C, D). The results of RI and LI showed that the RI of rats in the Model group was significantly increased (*P* < 0.01), but there was no significant effect on LI and RI in the administered groups (Fig. 1E, F). Detailed data on body weight and organ indices of rats are given in Supplementary Material (Supplementary Table 1).

### Effect of SSTL on H&E staining of prostate tissue in BPH rats

**Fig. 2:**
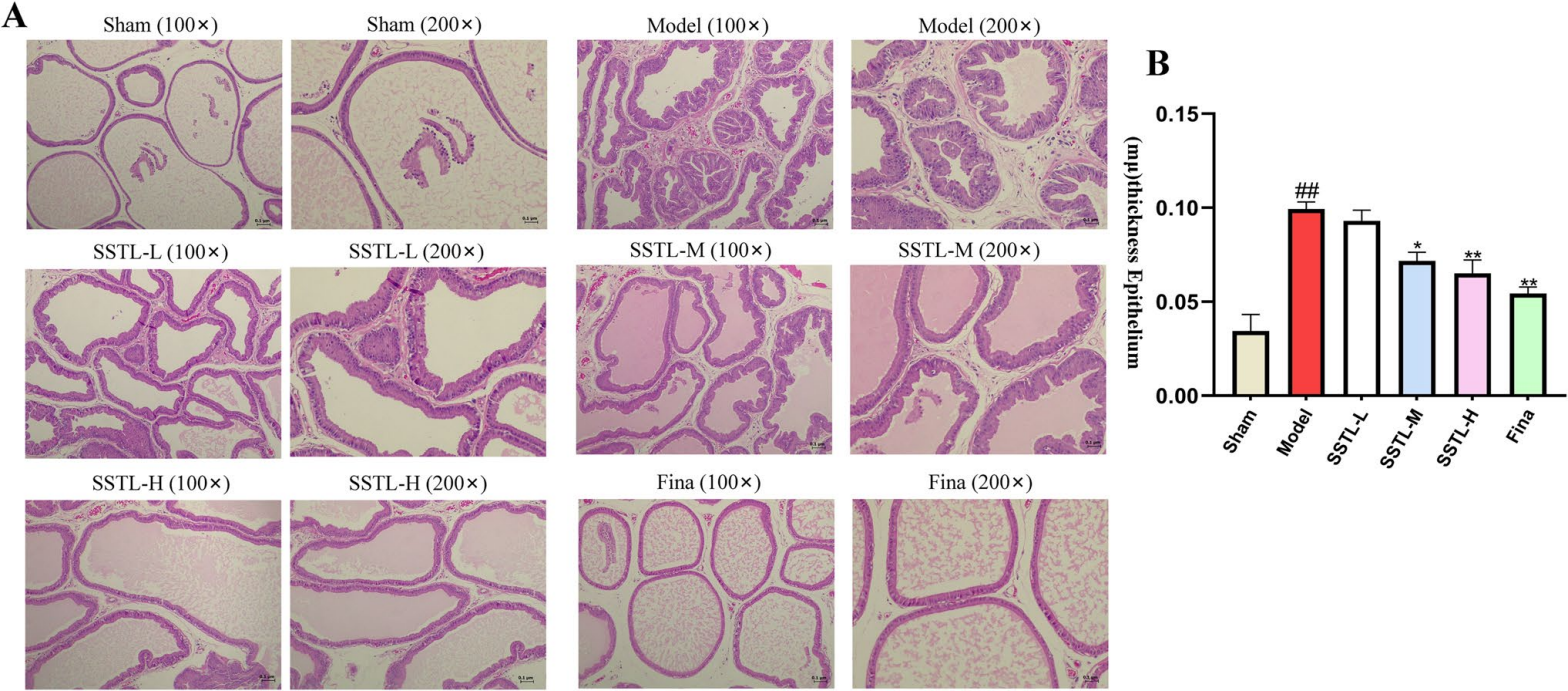
Effect of SSTLC on H&E staining of prostate tissue in BPH rats. **A** Representative H&E staining of prostate tissue, Each group of rats was observed under 100 × and 200 × field of view; **B** Thickness of prostate epithelium in each group of rats (*n* = 6)

In the Sham group, the epithelial cells of the rat prostate were seen to be neat, with no inflammatory infiltration or mesenchymal hyperplasia. In the Model group, the glands were seen to protrude into the lumen of the gland, with an irregular shape of the epithelium, and the mesenchymal stroma was markedly hyperplastic (Fig. 2A). Quantification of prostate epithelial thickness showed that significant epithelial cell thickening was seen in the Model group (*P* < 0.01), and epithelial thickness was significantly lower in the SSTL-M, H and Fina groups (*P* < 0.01 or *P* < 0.05) (Fig. 2B).

### Effect of SSTL on Masson staining of prostate tissue from BPH rats

**Fig. 3:**
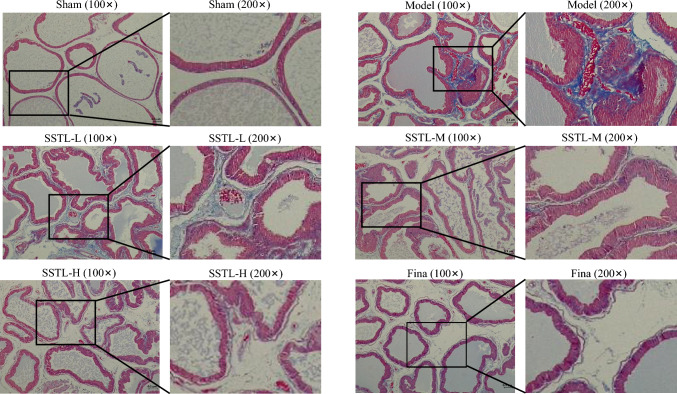
Effect of SSTLC on Masson staining of prostate tissue from BPH rats. Masson staining of rats in each group, each group of rats was observed under 100 × and 200 × field of view

Collagen deposition in the prostate of BPH rats was observed through Masson staining and collagen and fibers in the prostate tissue were stained blue. Collagen deposition in the prostate of BPH rats was observed by Masson staining, and collagen and fibers in the prostate tissue were stained blue. The degree of collagen deposition in the rat prostate tissue was significantly reduced in the SSTL-L, M and H groups and the Fina group compared with the Model group, and the walls of the vesicles were thinner, and there was secretion stained blue evenly distributed in the vesicles (Fig. 3).

### Effects of SSTL on sex hormones (T, DHT) in serum and prostate tissue of BPH rats

**Fig. 4:**
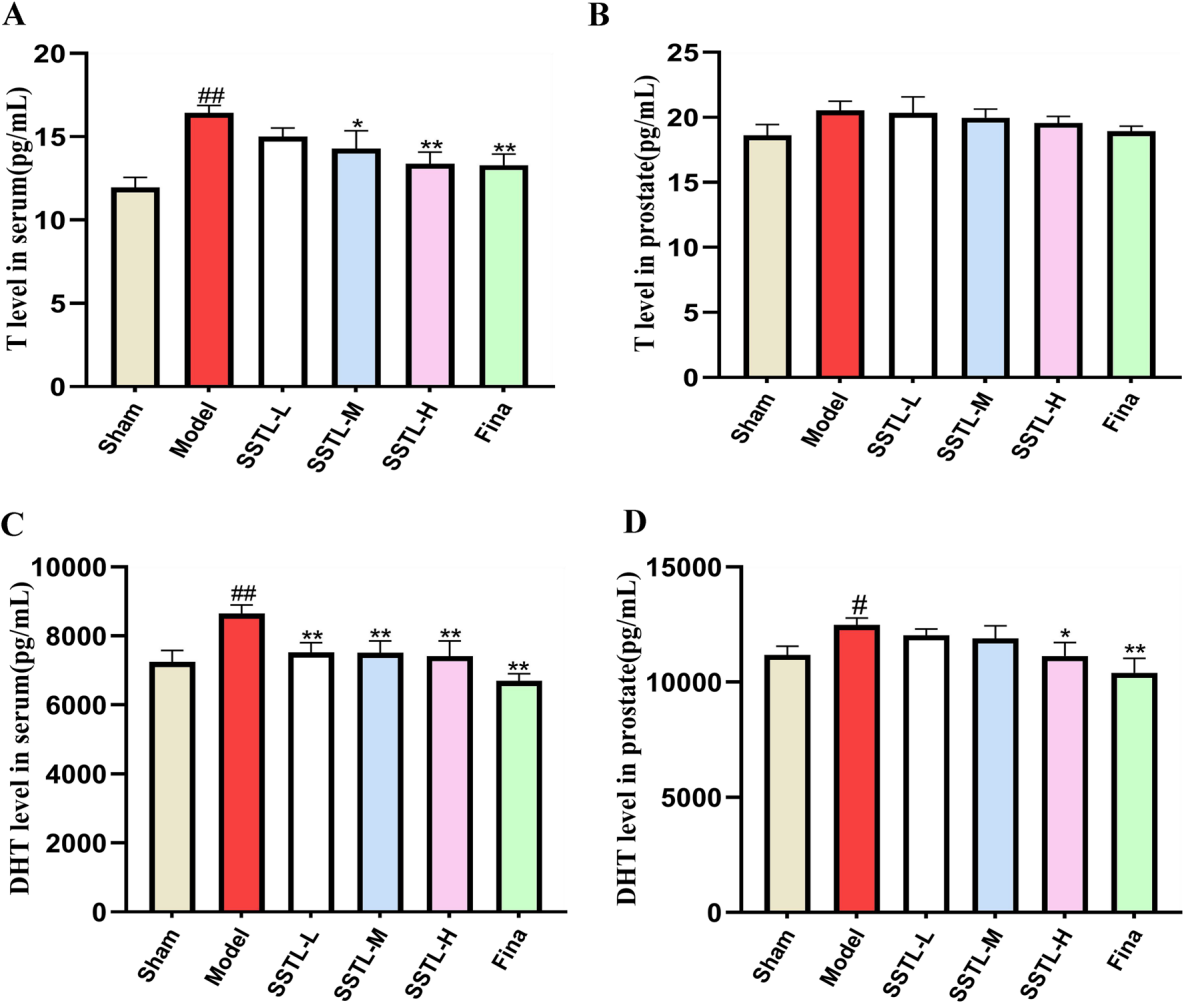
The expression levels of T, DHT in serum and prostate tissue. **A** T level in serum; **B** T level in prostate; **C** DHT level in serum; **D** DHT level in prostate; Values are presented as the means ± SD. #*P* < 0.05, ##*P* < 0.01 with the Sham group; *P < 0.05, **P < 0.01 with the Model group (*n* = 6)

The level of T in the serum of rats in the Model group was significantly higher, (*p* < 0.05) T levels in serum were significantly decreased in the SSTL-M and H groups and the Fina group. (*P* < 0.05 or *P* < 0.01) There were no significant changes in the T content of the various subgroups in the prostate tissue (Fig. 4A, B). DHT levels in serum and prostate tissues of rats in the Model group were significantly higher (*P* < 0.05 or *P* < 0.01), DHT levels in serum in the SSTL-L, M and H groups and the Fina group were significantly lower (*P* < 0.05) than those in the Model group, DHT in prostate tissue was significantly decreased in the SSTL-H group and the Fina group compared with the Model group (*P* < 0.05 or *P* < 0.01) (Fig. 4C, D).

### Effects of SSTL on inflammatory factors (TNF-α, IL-1β) in serum and prostate tissue of BPH rats

**Fig. 5:**
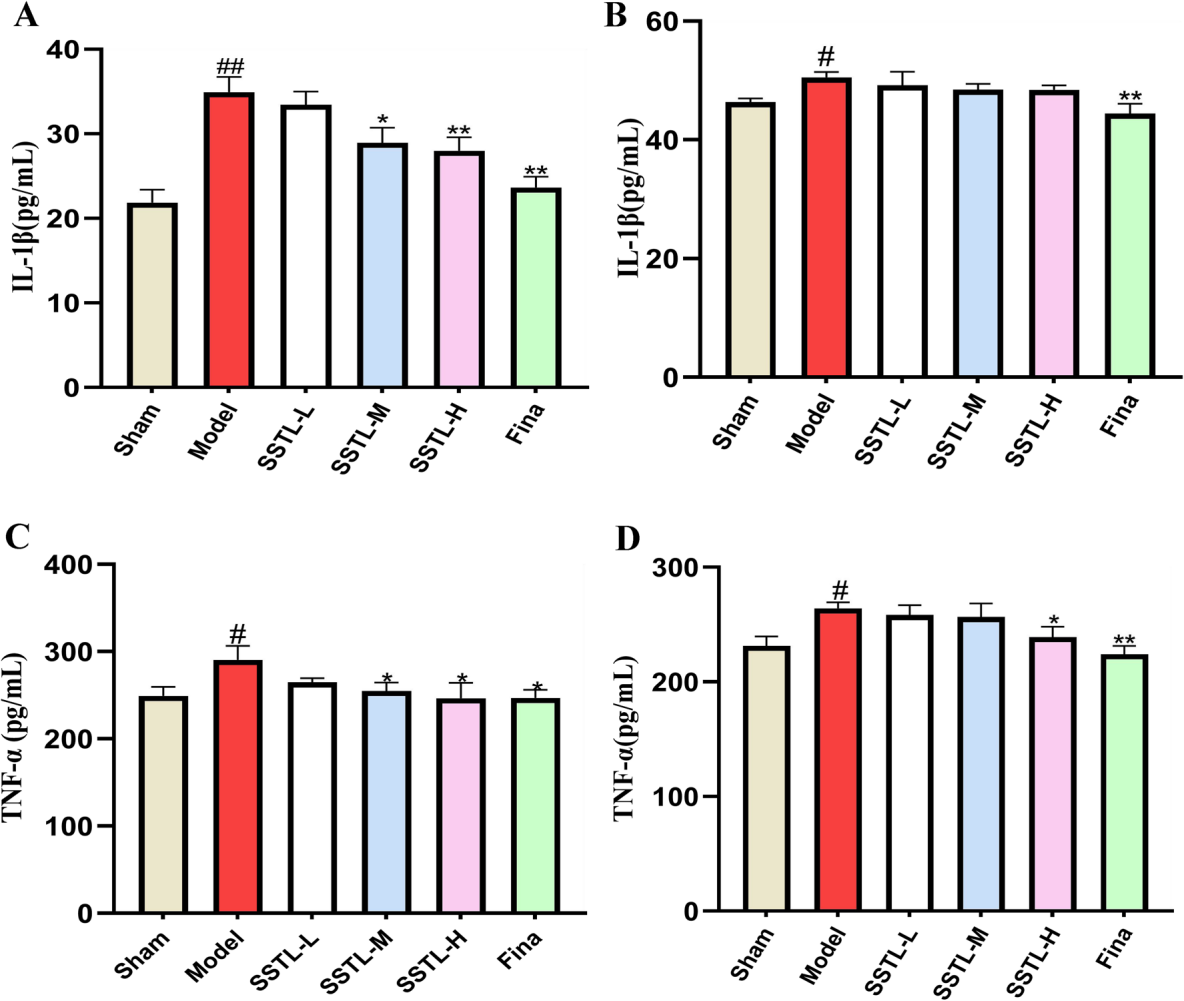
The expression levels of IL-1β, TNF-α in serum and prostate tissue. **A** IL-1β level in serum; **B** IL-1β level in prostate; **C** TNF-α level in serum; **D** TNF-α level in prostate; Values are presented as the means ± SD. #*P* < 0.05, ##*P* < 0.01 with the Sham group; *P < 0.05, **P < 0.01 with the Model group (*n* = 6)

The serum levels of IL-1β increased significantly in rats in the Model group (*P* < 0.01) and decreased significantly in rats in the SSTL-M, H and Fina groups, (*P* < 0.01 or *P* < 0.05). The content of IL-1β in the prostate tissues of rats in the Model group increased (*P* < 0.05), and SSTL had no significant effect on IL-1β in the prostate tissues. The content of IL-1β in the prostate tissues of the Fina group decreased significantly (*P* < 0.01) (Fig. 5A, B). The serum levels of TNF-α increased in the Model group (*P* < 0.05) and decreased in the SSTL-M, H and Fina groups (*P* < 0.05). TNF-α content in prostate tissues increased in the Model group (*P* < 0.05), SSTL-L and M groups had no significant effect on TNF-α in prostate tissues, and TNF-α content in prostate tissues decreased significantly in the SSTL-H and Fina groups (*P* < 0.01 or *P* < 0.05) (Fig. 5C, D).

### Detection of antioxidant enzyme activity and lipid peroxidation products

**Fig. 6:**
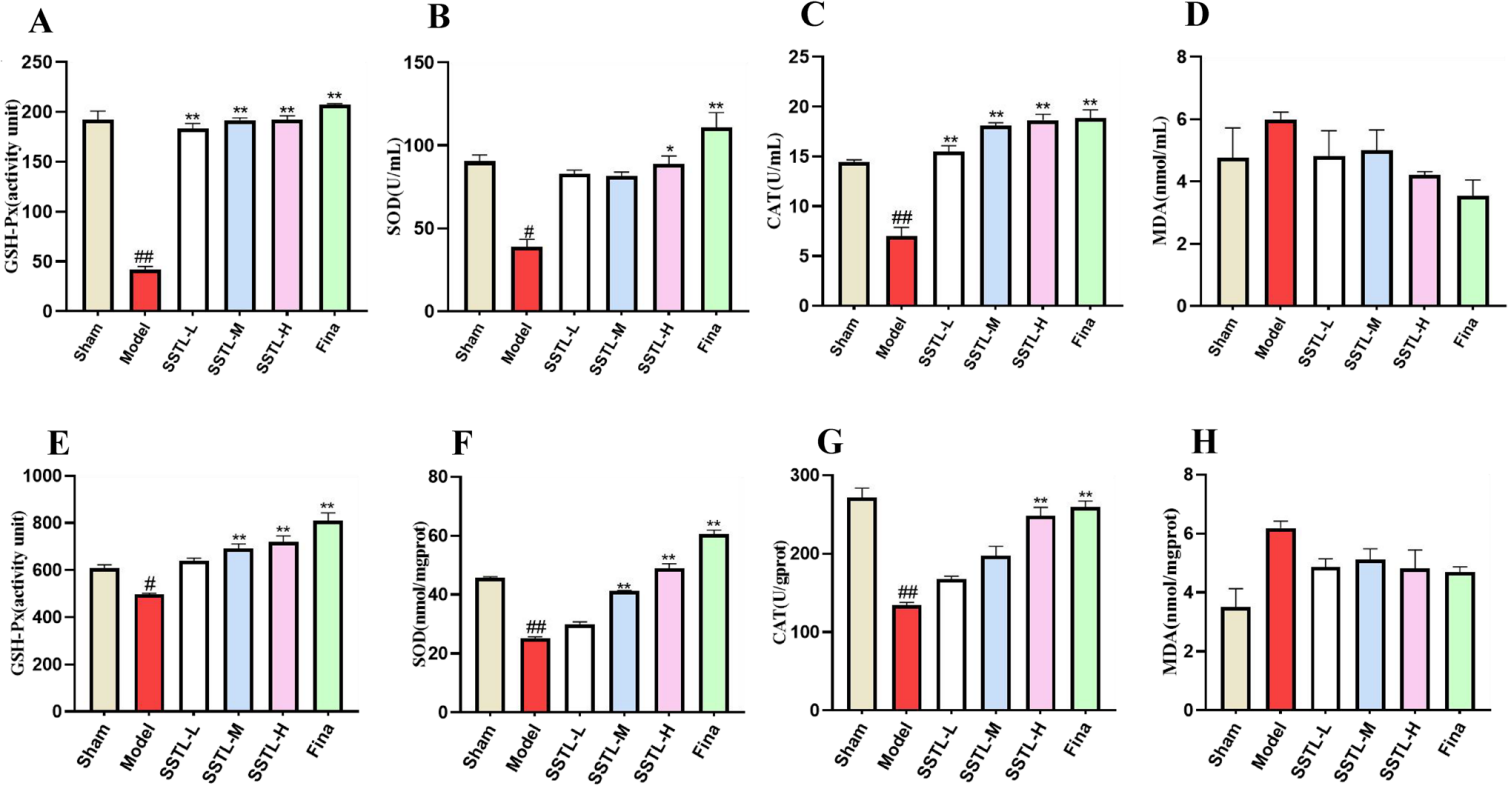
The expression levels of antioxidant enzyme activity and lipid peroxidation products. **A** GXH-Px level in serum; **B** SOD level in serum; **C** CAT level in serum **D** MDA level in serum; **E** GXH-Px level in prostate; **F** SOD level in prostate; **G** CAT level in prostate **H** MDA level in prostate; Values are presented as the means ± SD. #*P* < 0.05, ##*P* < 0.01 with the Sham group; *P < 0.05, **P < 0.01 with the Model group (*n* = 6)

The degree of oxidative stress in prostate tissue is positively correlated with BPH and prostate inflammation. SOD, CAT and GSH-Px have antioxidant activities, the final stabilized product of lipid peroxidation is MDA, which can be used as a marker of oxidative stress. The serum levels of SOD, GSH-Px and CAT were significantly lower in the Model group (*P* < 0.05 or *P* < 0.01), and the levels of GSH-Px and CAT were significantly higher in the SSTL-L, M, H, and Fina groups (*P* < 0.01). SOD activity was significantly increased in the SSTL-H and Fina groups (*P* < 0.05 or *P* < 0.01) (Fig. 6A–C). SSTL had no significant effect on MDA in rat serum (Fig. 6D).

The levels of SOD, CAT, and GSH-Px in the prostate tissues of rats in the Model group were significantly reduced (*P* < 0.01), GSH-Px, SOD levels were significantly higher in SSTL-M, H and Fina groups (*P* < 0.01). CAT was significantly elevated in the SSTL-H and Fina groups (*P* < 0.05) (Fig. 6E–G). SSTL had no significant effect on MDA activity in rat prostate tissue (Fig. 6H).

### ROS staining of prostate tissue was observed by frozen section

**Fig. 7:**
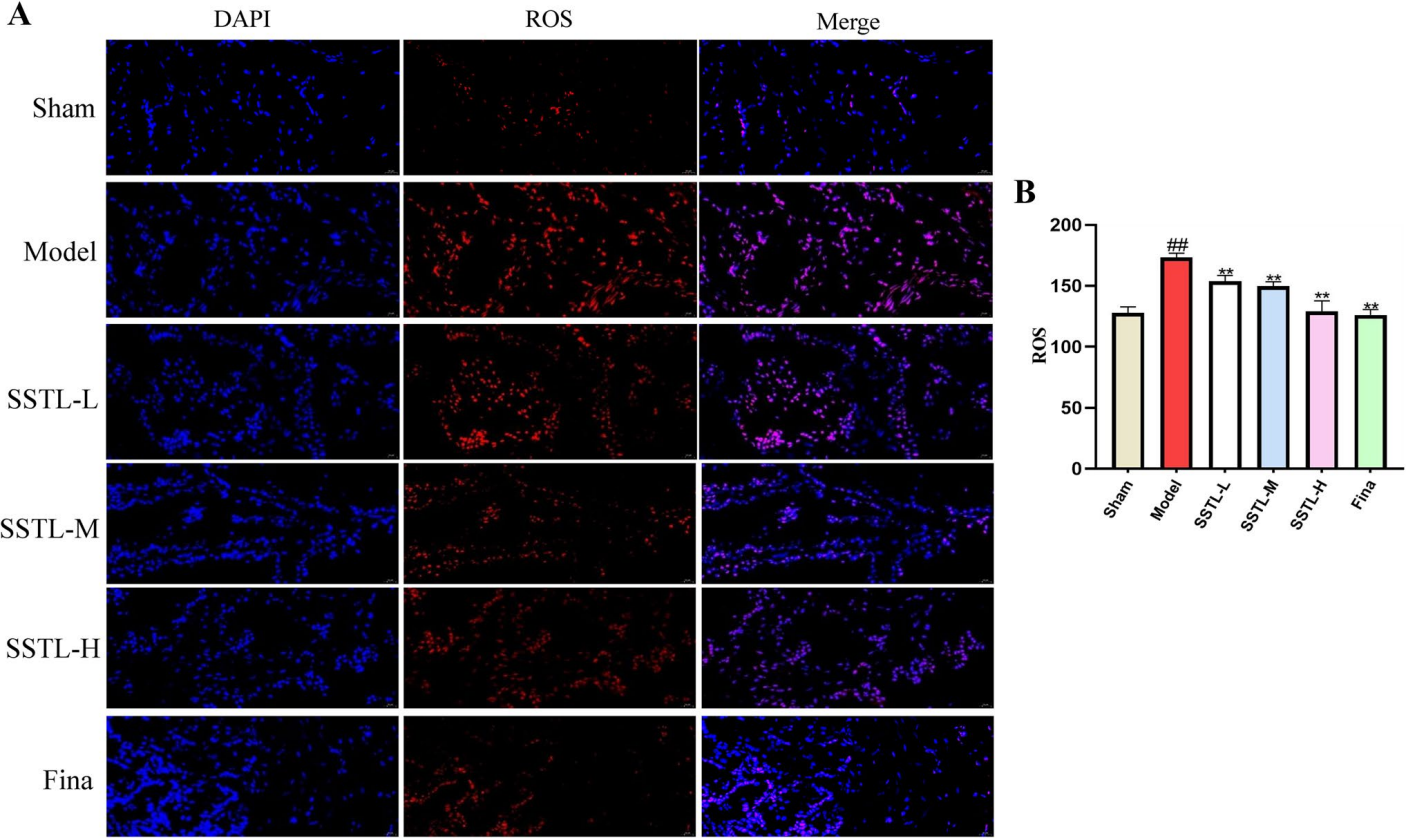
ROS immunofluorescence of prostate tissue. The ROS immunofluorescence signal is red and the DAPI immunofluorescence signal is blue. **A** Immunofluorescence staining for ROS in each group of rats; **B** Quantification of ROS immunofluorescence in each group of rats. Values are presented as the means ± SD. #*P* < 0.05, ##*P* < 0.01 with the Sham group; *P < 0.05, **P < 0.01 with the Model group (*n* = 6)

Oxidative stress may be caused when there is an imbalance between reactive oxygen species and antioxidants, which can damage to tissue proteins, DNA, and mRNA and increase the risk of increased cell proliferation, leading to BPH. The mean fluorescence of ROS in prostate tissues was significantly higher in the Model group compared to Sham (*P* < 0.01), and the level of ROS in prostate tissues was significantly lower in the SSTL-L, M and H groups, as well as the Fina group (*P* < 0.01) (Fig. 7).

### Effect of SSTL on immunohistochemistry of NLRP3 protein in BPH rats

**Fig. 8:**
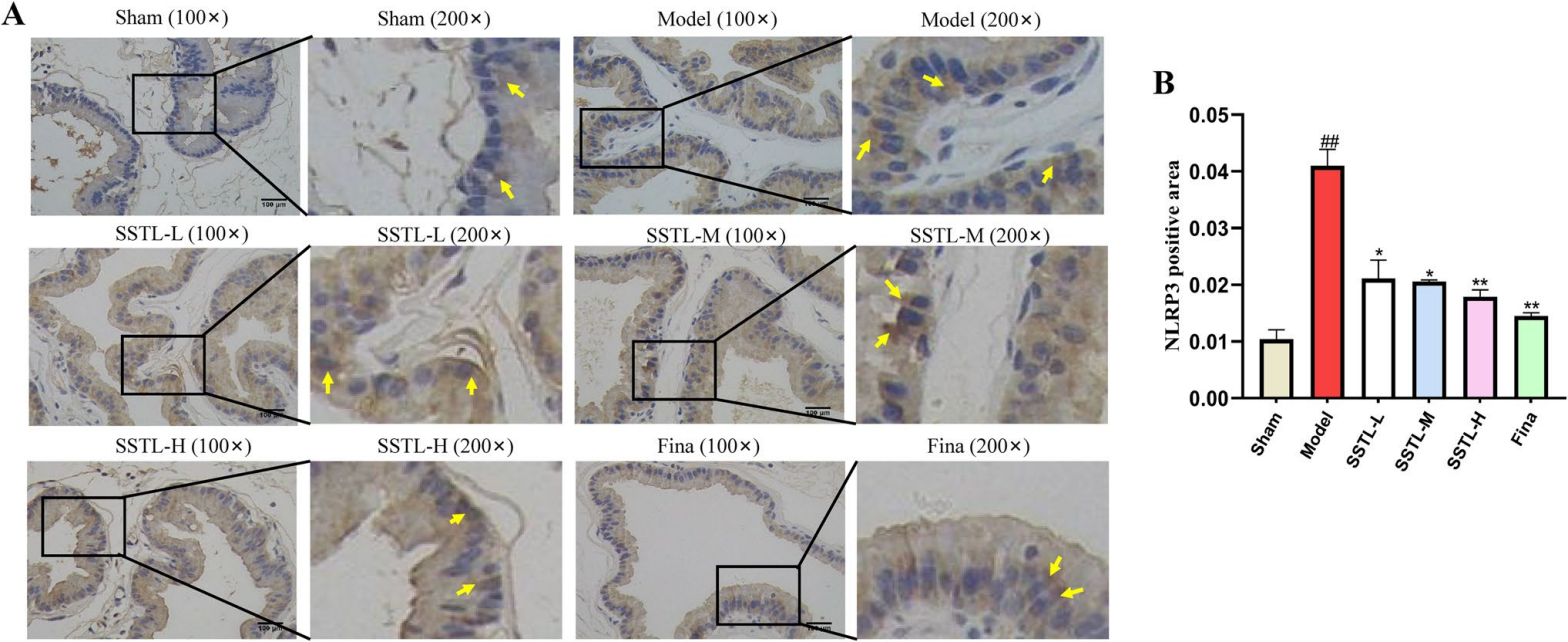
Immunohistochemistry of NLRP3 protein expression in various groups of rats. **A** Photomicrographs of immunohistochemical for the effects of SSTL capsule on the expressions of NLRP3, the yellow arrows indicate the expression of NLRP3, each group of rats was observed under 100 × and 200 × field of view; **B** Quantitative results of NLRP3-positive regions. Values are presented as the means ± SD. #*P* < 0.05, ##*P* < 0.01 with the Sham group; *P < 0.05, **P < 0.01 with the Model group (*n* = 6)

To determine the correlation between NLRP3 inflammatory vesicles and prostate hyperplasia, IHC was used to detect the localized expression of NLRP3 in prostate tissues, and brown was positive for expression (Fig. 8A). Quantitative results showed that the Sham group had less NLRP3 expression in prostate tissues; Model group rats had higher NLRP3 expression in prostate tissues (*P* < 0.01), and the expression level of NLRP3 inflammatory vesicles in prostate tissues of rats in SSTL-L, M, H groups and Fina group significantly decreased after drug administration (*P* < 0.01 or *P* < 0.05) (Fig. 8B).

### Effect of SSTL on the expression of ROS/NLRP3-related proteins in prostate tissues of BPH rats

**Fig. 9:**
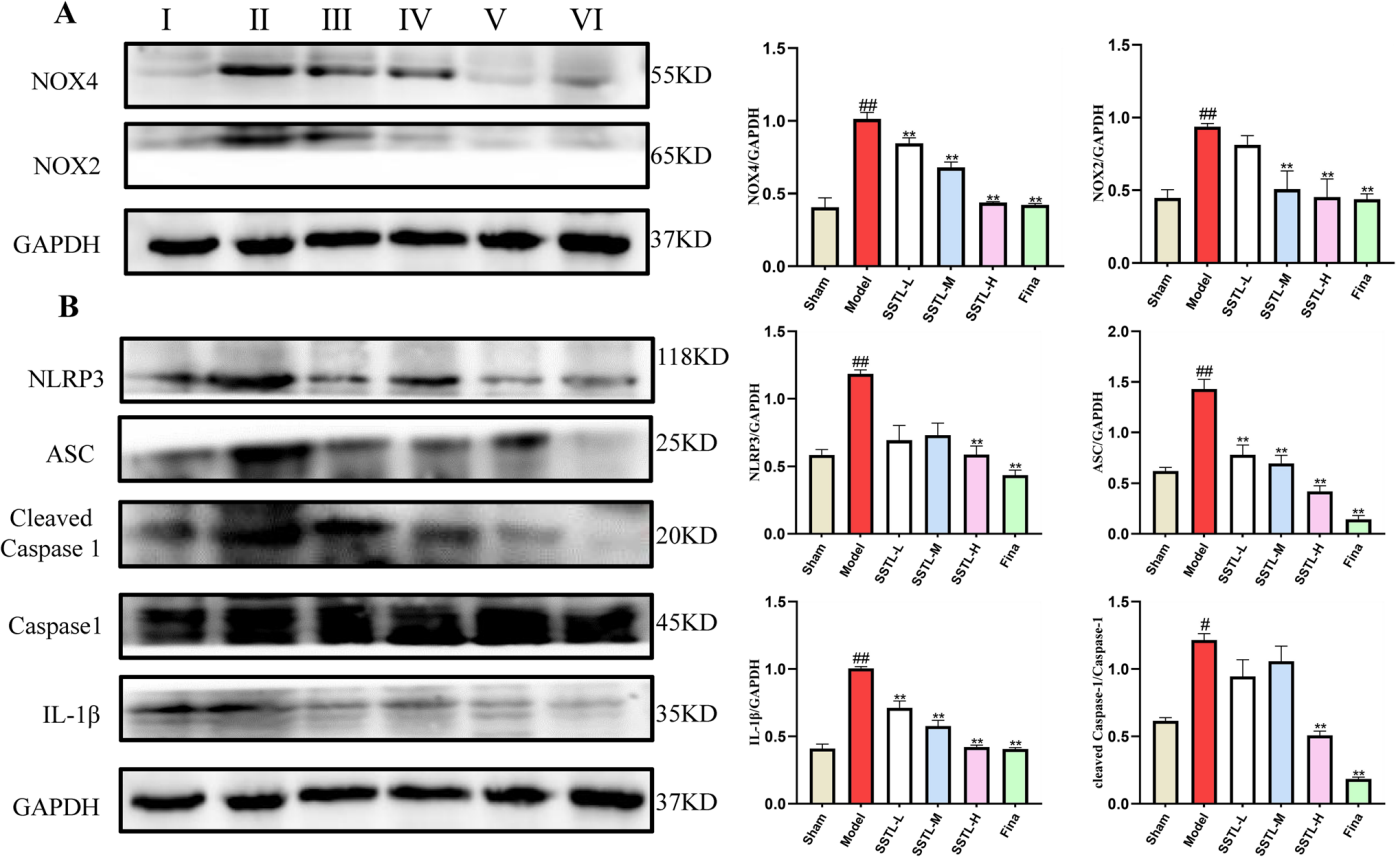
Effect of SSTL capsule on NOX-2, NOX-4 and NLRP3 signaling pathway. **A** Western blot analysis of NOX-2, NOX-4 and GAPDH expression in prostate tissues; **B** Western blot analysis of NLRP3, ASC, Cleaved Caspase-1, Caspase-1, IL-1β and GAPDH expression in prostate tissues. I: Sham group II: Model group III SSTL-L group IV: SSTL-M group V: SSTL-H group VI: Fina group. Values are presented as the means ± SD. ^#^*P* < 0.05, ^##^*P* < 0.01 with the Sham group; *P < 0.05, **P < 0.01 with the Model group (*n* = 3)

Protein expression of NOX4 and NOX2 was significantly higher in the Model group (*P* < 0.01), NOX4 protein expression was significantly lower in the SSTL-L, M, H and Fina groups (*P* < 0.01), and NOX2 protein expression was significantly lower in the SSTL-M, H and Fina groups (*P* < 0.01) (Fig. 9A). These results suggest that the protein expression of NOX2 and NOX4 is elevated in prostate tissues of BPH rats. Additionally, it indicates that SSTL may reduce oxidative stress injury by decreasing the expression of NOX2 and NOX4 proteins. The protein expression of NLRP3, ASC, Cleaved-caspase 1, and IL-1β in the prostate tissues of rats in the Model group was significantly elevated (*P* < 0.01), and the SSTL-H and Fina groups were able to significantly reduce the expression of NLRP3 (*P* < 0.01). The expression of ASC in the SSTL-L, M, H, and Fina groups was significantly reduced by the expression of ASC (*P* < 0.01); SSTL-L, M, H and Fina groups significantly reduced the protein expression of IL-1β (*P* < 0.01); SSTL-H and Fina groups significantly reduced the protein expression of Cleaved-caspase 1 (*P* < 0.01).(Fig. 9B) The above results suggested that the expression of NLRP3 and related proteins was up-regulated in the prostate tissues of testosterone propionate-induced BPH rat model, and SSTL could inhibit the upregulation of NLRP3 inflammatory vesicles and related proteins.

## Discussion

BPH is prostatic hypertrophy caused by the rapid proliferation of stromal and epithelial cells [20]. Hyperproliferation of the prostate causes various histologic changes such as an increase in epithelial thickness and infiltration of epithelial tissue into the ductal region, which can lead to urinary disorders including an LUTS and BOO [21]. Most patients with BPH are over 50 years of age and BPH often induces clinical symptoms such as frequent urination, lack of voiding, increased frequency of nocturia, and urinary urgency, all of which seriously affect the quality of life for patients [12]. The exact etiology of BPH is unclear but androgen/estrogen imbalance, inflammation, and overproduction of growth factors are believed to play key roles in the development of BPH, and oxidative stress is considered important in the development of BPH [11, 22]. Oxidative stress can be broadly defined as an imbalance between oxidant production and antioxidant capacity, which in turn causes ROS production [23]. NOX4 has been described as a novel source of ROS induction in macrophages, and it has been reported that NOX4-derived ROS can promote BPH [24]. ROS can lead to cellular damage through multiple pathways, including the release of pro-inflammatory cytokines, depletion of antioxidants and activation of pro-inflammatory signaling pathways [25]. Oxidative stress injury and activation of the NLRP3 inflammasome play an important roles in the development of fibrotic diseases. The NLRP3 inflammasome and the proinflammatory cytokines it produces have been investigated in many inflammatory diseases such as atherosclerosis, microbial infections, and chronic inflammatory diseases [16, 26]. The NLRP3 inflammatory vesicle is a complex composed of the core protein NLRP3, the junction protein apoptosis-associated speck-like protein, ASC, and the effector protein Caspase-1 [27]. It has been shown that the inflammasome and its downstream inflammatory factors IL-18 and IL-1β are expressed in prostate tissues in a rat model of BPH [17], Additionally increased ROS is a classical mechanism for inducing activation of the NLRP3 inflammasome, which plays an important role in fibrotic diseases [28]. Fibrosis develops gradually in a repetitive cycle of inflammation and oxidative stress-induced damage and repair. Increased oxidative stress in BPH tissue is positively correlated with prostate weight and prostate inflammation. Therefore, reducing the inflammatory response and oxidative stress is one of the effective treatments for BPH [29].

In the present study, the BPH rat model was induced in SD rats by disruption of sex hormone levels in vivo using denudation combined with subcutaneous injection of TP and SSTL administration was performed after successful modeling. For the pharmacodynamic effects of SSTL, PI was used as a measure of the BPH animal model [26], and SSTL was able to reduce the prostate index in BPH rats. Increased collagen deposition and epithelial thickness in the prostate gland jointly promote the EMT process and thus regulate the occurrence of BPH. Irregular shape of the prostate gland, a significant increase in epithelial thickness, and aggravation of collagen deposition were observed in the Model group of rats can be observed through H&E staining and Masson staining, However treatment with SSTL improved the epithelial thickness of the rat prostate and reduced collagen deposition in the rat prostate tissue. The imbalance between T and DHT is one of the main pathophysiological factors of BPH [30], DHT and T are involved in prostate hyperplasia and play a crucial role in prostate growth and development. SSTL can reduce the levels of T and DHT in rats, which suggests that SSTL has a positive therapeutic effect on BPH rats.

For the mechanism of action of SSTL, ROS immunofluorescence showed that ROS content was elevated in the prostate tissues of BPH rats, and SSTL effectively reduced the content of ROS in the prostate tissues of rats. The kit assay showed that SSTL exerted antioxidant stress response by inhibiting the expression of MDA, SOD, CAT, and elevating the activity of SOD, and the expression of TNF-α and IL-1β was elevated in Model rats, and the release of the inflammatory factors, TNF-α and IL-1β, was reduced by the administration of SSTL. These results suggest that oxidative stress injury and release of inflammatory factors are present in the rat model of BPH induced by TP, and that SSTL can reduce collagen deposition by decreasing inflammatory factors and oxidative stress, which in turn alleviates prostate hyperplasia. In one experimental study, in a rat model of BPH, prostate inflammation was found to be mediated by NLRP3 inflammasome-induced caspase-1 release and downstream release of TNF-α and IL-1β [31]. The results of this experimental study revealed that NOX2, NOX4, NLRP3 inflammatory vesicles and the expression of Caspase-1 and IL-1β were upregulated in prostate tissues in the rat model of BPH induced by TP, suggesting that NOX4 and NOX2-mediated production of excess ROS plays an important role in the activation of NLRP3. SSTL ameliorates BPH by decreasing protein expression of NOX4, NOX2, NLRP3 inflammatory vesicles and Caspase-1.

## Conclusions

In this study, we investigated the pharmacological effects of SSTL and the pathogenesis of BPH, and the pharmacodynamic effects and molecular mechanisms of SSTL on BPH were investigated in a rat model of BPH caused by debulking combined with subcutaneous injection of TP, with prostate index, histopathologic sections, inflammatory factors, oxidative stress, and NLRP3 inflammatory vesicles were used as detection indexes. The results indicated that SSTL was able to reduce the PI, improve the epithelial thickness of the prostate, reduce the collagen deposition in the prostate tissue of rats with BPH, and reduce the levels of T and DHT in rats. Mechanistically, SSTL improves oxidative stress injury by reducing NOX2, NOX4, and ROS. It also regulates NLRP3 inflammatory vesicles and attenuates inflammatory factors (IL-1β, TNF-α) to reduce inflammation and oxidative stress injury to play a therapeutic role in the treatment of BPH. This suggests that SSTL has a good therapeutic effect on the BPH rat model, which provides a theoretical basis for the clinical treatment of BPH with SSTL.

### Supplementary Information

Below is the link to the electronic supplementary material.

Supplementary file1 (DOCX 14 kb)

## Data Availability

The datasets used and/or analyzed during the current study are available from the corresponding author on reasonable request.
