# Supplementary material for: Shuangshi Tonglin Capsule treats benign prostatic hyperplasia through the ROS/NLRP3 signaling pathway
Source: Int Urol Nephrol. 2023 Dec 1;56(4):1259–71. doi: 10.1007/s11255-023-03874-w (PMC10923981; doi:10.1007/s11255-023-03874-w)
Supplement: Supplementary file 1 — Supplementary file1 (DOCX 14 kb) [file 11255_2023_3874_MOESM1_ESM.docx]

Supplementary Table1: Body weight, organ wet weight and organ index of SD rats.

|  | Rat weight(g) | Liver weight (mg) | Renal weight (mg) | Prostate weight (mg) | LI (%) | RI(%) | PI(%) |
| --- | --- | --- | --- | --- | --- | --- | --- |
| Sham | 330.02±33.44 | 9725±54.62 | 1201±31.09 | 788±27.65 | 2.947±0.217 | 0.364±0.041 | 0.239±0.051 |
| Model | 327.49±27.89 | 9636±66.73 | 1428±34.26## | 2125±30.12^##^ | 2.941±0.209 | 0.437±0.047^##^ | 0.649±0.092^##^ |
| SSTL-L | 308.14±21.79 | 9021±59.16 | 1352±33.21 | 1776±29.82 | 2.751±0.199 | 0.439±0.028 | 0.577±0.081 |
| SSTL-M | 336.66±22.51 | 9549±61.48 | 1323±35.87 | 1846±31.97 | 2.842±0.227 | 0.394±0.024 | 0.548±0.034 |
| SSTL-H | 325.62±21.28 | 8869±65.42 | 1319±40.06 | 1669±34.18* | 2.729±0.231 | 0.406±0.048 | 0.512±0.086* |
| Fina | 352.52±28.03 | 9648±59.22 | 1281±39.02* | 1701±33.27* | 2.737±0.224 | 0.364±0.052* | 0.483±0.079* |

Values are presented as means ± SEMs. ^#^P<0.05, ^##^P<0.01 compared with the Sham group; * P <0.05, ** P <0.01 compared the Model group (n=6)
